# A Smart Capsule System for Automated Detection of Intestinal Bleeding Using HSL Color Recognition

**DOI:** 10.1371/journal.pone.0166488

**Published:** 2016-11-30

**Authors:** Panpan Qiao, Hongying Liu, Xueping Yan, Ziru Jia, Xitian Pi

**Affiliations:** 1 Key Laboratory of Biorheological Science and Technology of Ministry of Education, Bioengineering College, Chongqing University, Chongqing, PR China; 2 Key Laboratories for National Defense Science and Technology of innovative micro-nano devices and system technology, Chongqing University, Chongqing, PR China; 3 Chongqing Engineering Research Center of Medical Electronics, Chongqing, PR China; West Virginia University, UNITED STATES

## Abstract

There are no ideal means for the diagnosis of intestinal bleeding diseases as of now, particularly in the small intestine. This study investigated an intelligent intestinal bleeding detection capsule system based on color recognition. After the capsule is swallowed, the bleeding detection module (containing a color-sensitive adsorptive film that changes color when absorbing intestinal juice,) is used to identify intestinal bleeding features. A hue-saturation-light color space method can be applied to detect bleeding according to the range of H and S values of the film color. Once bleeding features are recognized, a wireless transmission module is activated immediately to send an alarm signal to the outside; an in vitro module receives the signal and sends an alarm. The average power consumption of the entire capsule system is estimated to be about 2.1mW. Owing to its simplicity, reliability, and effectiveness, this system represents a new approach to the clinical diagnosis of intestinal bleeding diseases.

## Introduction

Gastrointestinal (GI) hemorrhagic disease is a common clinical symptom [1–4], especially in the elderly patients. Clinical manifestations include vomiting and bloody diarrhea, and severe cases can cause shock or even death [5, 6]. Routine diagnosis methods for intestinal bleeding diseases, such as radionuclide scanning [7], digital subtraction angiography [8, 9], and endoscopic examination [10–12], generally are invasive or have substantial side effects, making physicians unlikely to administer them and patients unlikely to accept them [13, 14]. Capsule endoscopy represents a revolutionary breakthrough in noninvasive diagnosis of intestinal bleeding diseases [15]. The capsule endoscope enters the digestive tract by being simply swallowed, then obtains image information of each segment of the digestive tract and wirelessly sends the images to the outside where they can be reviewed by medical professionals [16]. Though the method is effective, there is sizeable burden placed on the physician–a rather excessive amount of image information (about 60000 images) [17, 18] must be scanned to identify bleeding lesion areas, and visual fatigue caused by long work hours may lead to human error [19]. With doctors’ large workloads and the complexity of image analysis, the cost of capsule endoscopy testing is very high [20]. In addition, the color of micro hemorrhages may blend into the background of the intestinal wall, making them difficult to identify in the images. Hong-ying LIU et al put forward a remote controlled capsule based on a red-green-blue (RGB) color space, which is not intuitive, to detect GI tract bleeding through directly observing blood and intestinal juice mixture [21].

This study proposes an intelligent intestinal bleeding detection capsule (IBDC) system based on color recognition of hue-saturation-light (HSL) color space in effort to ameliorate these problems in intestinal bleeding disease diagnosis. In this capsule, bleeding information is not obtained by observing blood and intestinal juice mixture directly, but by dying a color sensitive film, which is more obvious and accurate. The capsule system can send sound and light alarm signals automatically once detecting intestinal bleeding, and trace, small, and large amounts of blood correspond to different alarm signals, which makes diagnosis noninvasive, simple and greatly reduces the workload and cost of the tests. After the system registers an alarm signal, the bleeding point can be located in vitro according to specific needs. Capsule endoscopy can also be used to operate further tests as necessary, as well as targeted treatments. The capsule doesn’t use chemical methods to detect bleeding, because the chemical sensor is very sensitive and able to detect occult blood, however, our aim is to detect trace or more overt bleeding.

## 2. Materials and Methods

### 2.1 Capsule System Description and Evaluation

**Fig 1:**
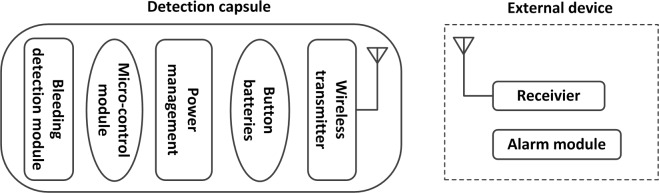
Functional block diagram of smart capsule system.

**Fig 2:**
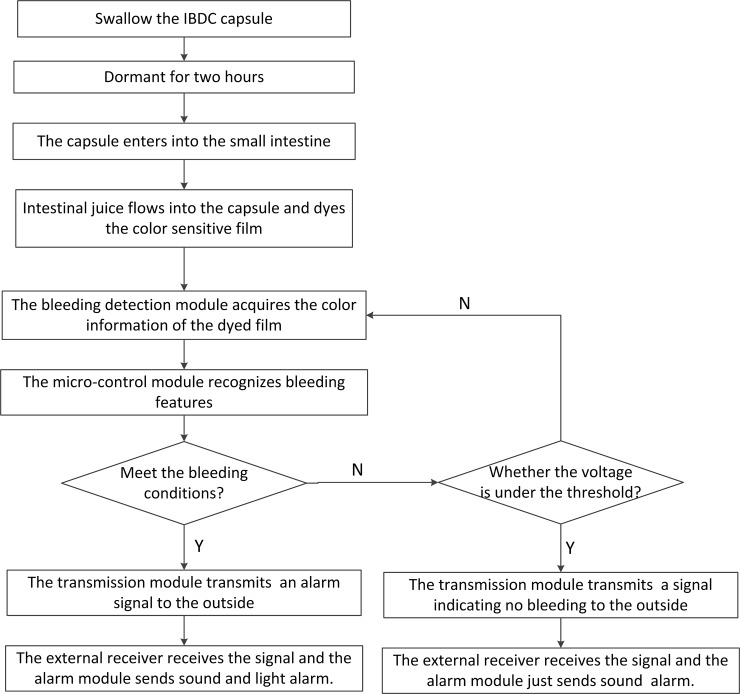
Work flow diagram of functional modules in capsule system.

The intelligent IBDC system includes a detection capsule, an external receiving module, and an alarm module. The smart capsule itself mainly consists of a bleeding detection module, micro-control module, wireless transmission module, and power management module. A functional block diagram of the capsule system is shown in Fig 1. The voltage lower threshold of the bleeding detection module is 2.7V and the voltage lower threshold of the transmission module is1.9V. Considering the situation that the bleeding detection module may also work under its lower threshold, the voltage of 2.4V was set to be the lower threshold of the capsule. The capsule is swallowed into the human body and moves forward along with GI peristaltic movement. After being swallowed, the capsule is dormant for two hours, and then the intestinal juice flows into the capsule and dyes the color sensitive film. If the operating voltage is above 2.4V, the bleeding detection module will acquire the color information of the dyed film and the micro-control module will recognize intestinal bleeding features. The detecting sensor works every 5s, sleeping for 5s and working for 0.5s. Once a bleeding feature is recognized, the wireless transmission module is activated immediately to send an alarm signal to the outside. The external receiver receives the signal and the alarm module sends sound and light alarm. If there is no bleeding, the capsule again goes dormant before beginning another color collection and identification process five seconds later. If the operating voltage is under 2.4V and no bleeding was detected, the wireless transmission module will also be activated and send a signal to the outside. The external receiver receives the signal and the alarm module just sends sound alarm. Work flow diagram of functional modules in capsule system is shown in Fig 2.

The bleeding detection module is the key component of the smart IBDC. Whether or not it can identify color information effectively determines the feasibility of the entire capsule system. Under normal circumstances, the color of the intestinal juice is colorless or transparent yellow. If there is intestinal bleeding, however, the blood flowing in the intestine causes certain differences in color compared to healthy intestinal juice. The color sensor is used to acquire color information for the intestinal juice and extract corresponding color feature parameters. According to quick discrimination between color feature parameters of bleeding and non-bleeding areas, intestinal bleeding detection can be realized effectively.

**Fig 3:**
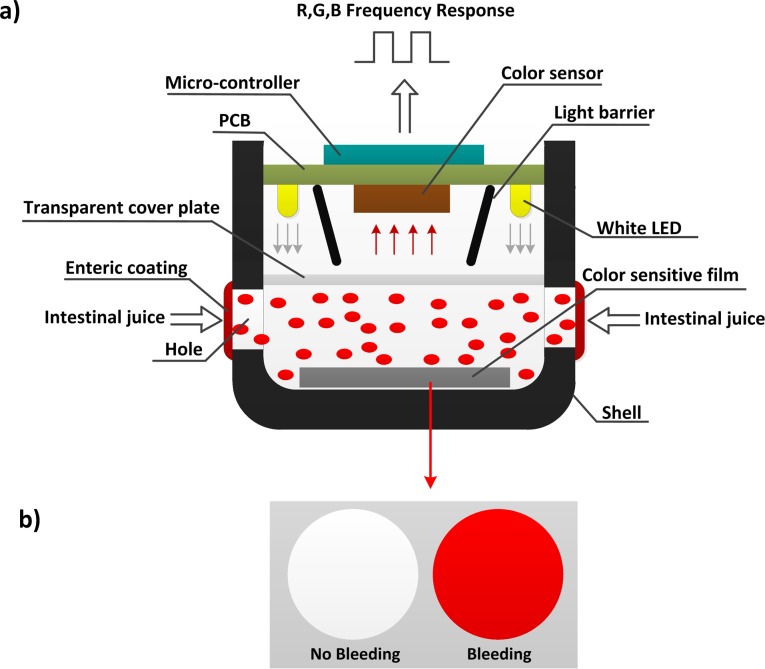
(a) Schematic diagram of bleeding detection module. (b) Color contrast of undyed and dyed color sensitive films.

A schematic diagram of the bleeding detection module is shown in Fig 3(A). This module consists of shell, enteric coating, adsorptive film, oval holes, a transparent cover, color sensor, black light barriers, LEDs, and a peripheral circuit. The shell of the capsule is comprised of non-transparent polycarbonate, which is non-toxic, innocuous, resistant to acidic and alkaline conditions, and has favorable mechanical properties and stability. The material of enteric coating is shellac, which is insoluble in gastric juice but quickly dissolved in a solution of above pH6.4. The role of enteric coating is to prevent gastric juice from flowing into the capsule to dye the adsorptive film. The adsorptive film (9mm diameter, 1.5mm thickness), which has strong selective adsorption capability to hemoglobin, is attached to the inner wall of the capsule shell. The outer layer of the slice is medical nonwoven fabric and mid-embedded 330 anion exchange resin; the hydrophobic interaction between the resin and hemoglobin allows hemoglobin to be adsorbed on the resin[22–24]. When intestinal bleeding occurs, blood flows into the capsule more easily to dye the adsorptive film due to the resin. Two axisymmetric oval holes are designed in the front-end of the shell with long axes of 7mm and short axes of 2.5mm to allow intestinal juice in and out of the capsule smoothly. Electronic components are separated from intestinal juice by a transparent cover. The LEDs act as a light source, and black baffles are used to prevent the interference of incident light on both sides. The color sensor acquires reflected color information and sends it to the microcontroller for processing.

If there is no intestinal bleeding, the adsorptive film is white. If there is bleeding, the intestinal juice dyes the adsorptive film (Fig 3(B)). The color information acquired by the color sensor is different according to different amounts of blood detected, and the microcontroller analyzes the results based on this difference.

This paper adopts the color sensor TCS3200 (AMS-TAOS, USA). The sensor can convert the light intensity of trichromatic color components (red, green, blue) into square wave pulses with corresponding frequencies [25]. A microcontroller, PIC16F690 (PIC: Peripheral Interface Controller, Microchip Technology In, USA), with input capture functions is used to calculate the frequencies, and then the color is converted into trichromatic components. The color detection circuit contains two white light emitting diode (LED) lights for illumination.

Color information is detected by the TCS3200 color sensor in RGB color space, which is not intuitive [26], and its process of distinguishing color aberrations is nonlinear [27]; basically, RGB color space is not suitable for color recognition. HSL color space, however, is more intuitive and consistent with human visual characteristics [28], and is typically used in color recognition applications. The smart capsule was designed to convert trichromatic components of color into HSL color space to identify intestinal bleeding.

H (hue, H∈[0°, 360°]) is the range of the color that human can perceive. These colors distribute in a hue circle and each angle represents a color. S is the color saturation; the value of S (S∈ [0, 1]) describes the discrimination of color purity in the case of the same hue and lightness. [29]. Because L (light, L ∈[0, 1]) is not relevant to the color information, the smart capsule neglects the L parameter. The closer to 0 or 360 the H and the higher the S, the deeper red the color is [30].

**Fig 4:**
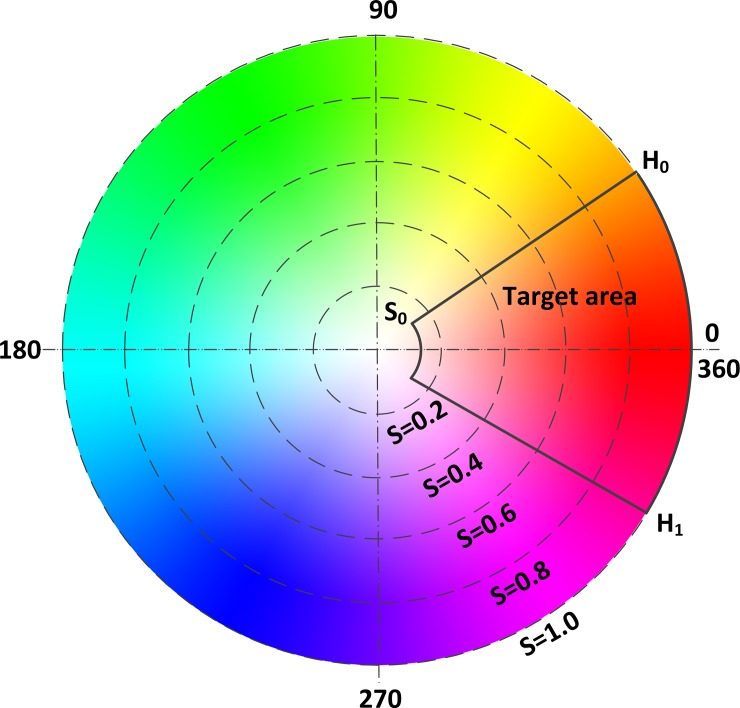
Target area of color features.

**Fig 5:**
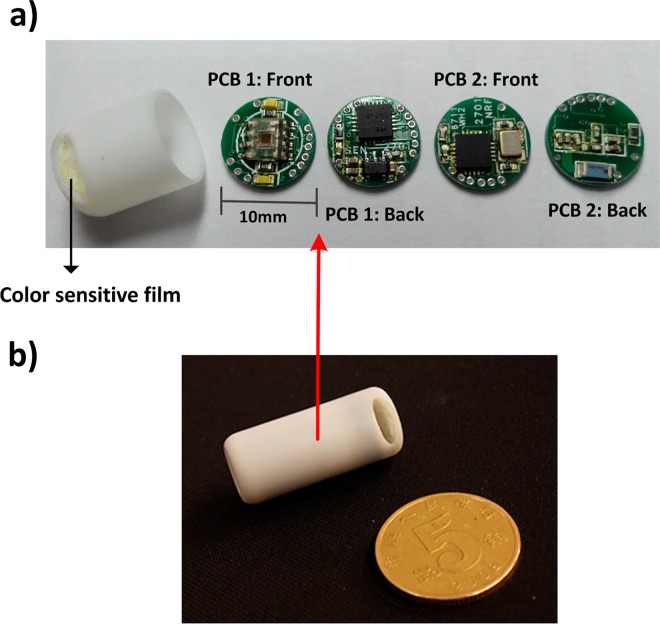
(a) Double-sided contained. From left to right: color sensor and LEDs, microcontroller and power management, wireless transmitter, antenna and impedance matching network. (b) Photograph of smart capsule prototype.

As mentioned above, H and S act as bleeding recognition characteristic parameters in the proposed system. The target area of color features is shown in Fig 4. Color feature parameters [H0, S0] ∪ [H1, S0] are set as critical conditions for bleeding recognition–if H and S fall in the target area shown in Fig 4, that is, H∈[0, H0] ∪ [H1, 360] and S∈ [S0,1], intestinal bleeding is confirmed. The bleeding detection module is depicted in Fig 5(A).

Timely response based on real-time data transmission is desirable and not all data will be transmitted to the outside [31–33]. Therefore, in this study, when test results meet the decision conditions of bleeding set by the program, the microcontroller triggers the wireless data transmitter module and a series of encoded signals are transmitted to the outside. The main aim in the area of wireless transmission is to achieve the maximum coverage in the area with the least energy consumption [33, 34]. However, because the extracorporeal receiving module is very close to the human body, the transmission distance does not need to be particularly far. This study adopted RF (Radio Frequency) devices without upper-layer protocol to achieve low power consumption and short-distance wireless data transmission functions. The carrier frequency was selected as 2.45GHz. The prototype includes a monolithic nRF24L01 RF transceiver chip (Nordic semiconductor, Norway), working at 2.4GHz-2.5GHz ISM (Industrial Scientific Medical) band, with a QFN (Quad Flat No-lead Package) package measuring only 4mm*4mm. The device is very well-suited to the strictly limited space inside the capsule system. When not performing data transmission, the nRF24L01 is set to power-down mode; when performing data transmission, the nRF24L01 is set to transmission mode.

A ceramic patch antenna, AN3216 (RainSun, Taiwan), using 2-pin SMD (Surface Mounted Devices) package and measuring 3.2mm*1.6mm, with characteristic impedance of 50Ω and return loss of 19.847dB [35], was also selected for our design. The antenna is very small and its emission efficiency is very high (2.45GHz). In the wireless transmission circuit, the antenna connects to the nRF24L01 RF chip interface through the impedance-matching network. There is also a 4-pin SMD package with frequency of 16MHz. Peripheral capacitance and inductance components comprise the SMD 0402 package. The wireless transmission circuit is completely integrated within a diameter of a 10mm double-sided PCB2 (printed circuit board) board shown in Fig 5(A).

The external device includes a wireless receiving module and an alarm module. An nRF24L01 RF chip and AN3216 antenna were also used in the wireless receiving module, and an alarm module, which includes a buzzer and three red LEDs, is integrated on the receiving module. When trace bleeding is detected, the buzzer sounds and one LED comes on. When a small amount of bleeding is detected, the buzzer sounds two LEDs come on, and when there is a large amount of bleeding, the buzzer sounds and all three LEDs come on. When the operating voltage is under 2.4V and no bleeding is detected, only the buzzer sounds. The alarm module connects to the output port of the wireless receiving module through a microcontroller. Once a signal sent by the smart capsule inside the body is received, the microcontroller signals the alarm module to alarm according to the specific encoded signal to alert the physician to the patient’s intestinal bleeding.

In order to allow the capsule to monitor the entire intestinal tract, button batteries ensure a stable power supply for 6~8 hours. We used two SR920SW silver oxide batteries (MAXELL, Switzerland) with diameter of 9.5mm, thickness of 2.05mm, nominal voltage of 1.55V, and nominal capacity of 40mAh [36]. Power regulator chip MAX1724 (Maxim, USA) was also selected, with output voltage of 3.0V, SOT23-5 package, and 2.75mm*1.9mm size. The magnetic switch is a two-pin normally closed reed.

The photograph of smart capsule prototype is shown in Fig 5(B). The smart capsule is preserved in a box filled with a permanent magnetic field in a specific direction. When not in use, due to the magnetic field, the magnetic switch is turned off and the capsule does not work. When in use, the capsule is removed from the box. As it escapes the magnetic field, the magnetic switch recovers the on state and the capsule begins to work.

### 2.2 Experimental Methods

An experiment was conducted to verify the bleeding detection effect and to determine bleeding recognition conditions of the proposed IBDC system. The double dilution method was applied in the experiment. Normal human blood was from a consenting adult male, with hemoglobin concentration of 152g/L. Protocols of this study was approved by Medical Ethics Committee of Chongqing. The volunteer signed the informed consent after the procedure of the study was explained in detail. The blood sample was diluted into different concentrations to simulate different amounts of intestinal bleeding. Blood samples were supplemented with an appropriate amount of heparin anticoagulant, shaken evenly, then supplied PBS buffer as a diluent. Ten sets of test solutions were prepared. Test solutions 1 and 2 simulated normal human intestinal juice (colorless and transparent yellow) without bleeding, and solutions 3–10 are blood sample solutions diluted into different multiples. As dilute ratio increased, the color variation of the solutions went from deep red→ red→ light red→ colorless.

To avoid the interference of external light, the entire testing process must take place in a closed black box. The signal output pin of color sensor was connected to a UT2025B digital oscilloscope, and two silver oxide button batteries provided power for the bleeding detection circuit.

**Fig 6:**
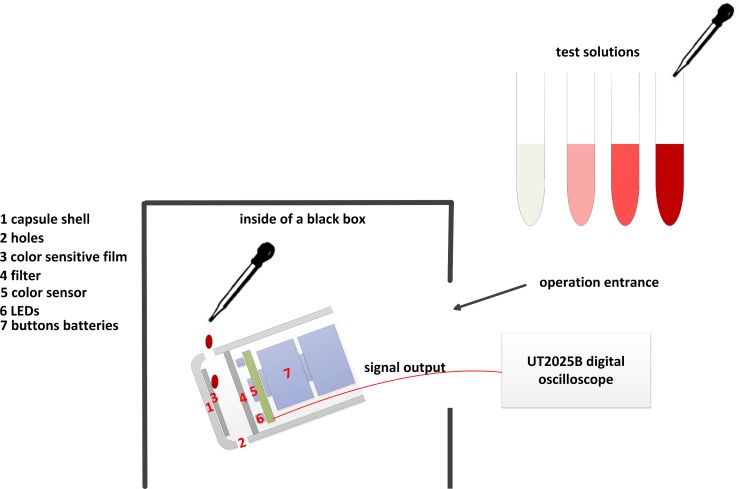
Diagram of operation process.

Fig 6 shows the operation process. The experiment was conducted as follows.

1. We tilted the encapsulated bleeding detection module to the appropriate degree and fixed it.
2. We connected the encapsulated module to the power, adjusted the lighting circuit voltage, and stabilized the lighting circuit current at 5mA.
3. We absorbed 1mL of Solution 1 with a dropper and dropped it onto the color sensitive film inside the bleeding detection module.
4. We selected the red filter and recorded the corresponding frequency value of the red component.
5. We selected the green filter and recorded the corresponding frequency value of the green component.
6. We selected the blue filter and recorded the corresponding frequency value of the blue component.
7. We replaced the color sensitive film.
8. Steps 3–7 were repeated ten times to record the dataset, then we calculated the corresponding mean frequencies $\bar{f_{R}}$, $\bar{f_{G}}$, and $\bar{f_{B}}$ of each color component.
9. Experiments of Solutions 2–10 were conducted similarly to obtain the corresponding mean frequencies of the dyed color-sensitive film.

An in vitro experiment was conducted to verify the wireless data transmitting and receiving capabilities of the IBDC system, as well as the effectiveness of the bleeding alarm. We set the initial H value as H0 = 0, the initial S value as S0 = 0, and bleeding recognition conditions as 3≤H≤25 and 0.3≤S≤0.8. When H and S met the set conditions, the buzzer sounded and the LEDs came on.

Three groups of sample solution were prepared and numbered 1–3. Solution 1 simulated intestinal juice with transparent yellow color, Solution 2 simulated colorless intestinal juice, and Solution 3 was human blood diluted 64 times.

As discussed above, the bleeding detection module, microcontroller, button batteries, and wireless transmitting module are encapsulated together. The wireless receiver and alarm module are powered by a regulated DC supply. During our experiment, only one red LED was used to indicate bleeding, so there was no discrimination between trace, small, or large amounts of blood.

First, 1mL of Solution 1 was absorbed with a dropper and dropped onto the color-sensitive film inside the bleeding detection module. Inside and outside power were then switched on. The status of the buzzer and the LED were then read and recorded. The color-sensitive film was then replaced and the procedure was repeated for Solutions 2 and 3.

## 3. Results and Discussion

### 3.1 Performance Testing Results

The test results of the experiment to verify the bleeding detection effect and to determine bleeding recognition conditions of the proposed IBDC system are shown in Table 1.

**Table 1:** Corresponding frequencies of sample solutions and H, S values.

Number i	Dilution ratio	${\bar{f}}_{Ri}$(kHz)	${\bar{f}}_{Gi}$(kHz)	${\bar{f}}_{Bi}$(kHz)	H (kHz)	S (kHz)
**1**	Intestinal juice 1	2.6483	3.2657	2.7727	46	0.33
**2**	Intestinal juice 2	3.0148	3.9876	4.4974	60	0.10
**3**	512	2.8477	3.7075	4.0711	41	0.20
**4**	256	2.7722	3.5482	3.8479	35	0.23
**5**	128	2.6774	3.3041	3.5226	28	0.27
**6**	64	2.5312	2.8258	2.9179	20	0.33
**7**	32	2.3521	1.9657	1.8391	14	0.42
**8**	16	2.1432	1.2072	1.4065	9	0.51
**9**	4	1.8243	0.7237	0.6862	4	0.60
**10**	1	1.7325	0.6087	0.5918	3	0.63

The white balance adjustment parameters of the color sensor in this system are $\bar{t_{R}}$ = 81.4097ms, $\bar{t_{G}}$ = 61.5481ms, and $\bar{t_{B}}$ = 54.0988ms. The corresponding RGB trichromatic components of each test solution after white balance adjustment can be obtained via Eqs (1–3).

$$R_{i}=\bar{f_{Ri}}\times \bar{t_{R}}\begin{array}{cc} & \left(i=1,2,\ldots \ldots ,10\right) \end{array}$$ (1)

$$G_{i}=\bar{f_{Gi}}\times \bar{t_{G}}\begin{array}{cc} & \left(i=1,2,\ldots \ldots ,10\right) \end{array}$$ (2)

$$B_{i}=\bar{f_{Bi}}\times \bar{t_{B}}\begin{array}{cc} & \left(i=1,2,\ldots \ldots ,10\right) \end{array}$$ (3)

**Fig 7:**
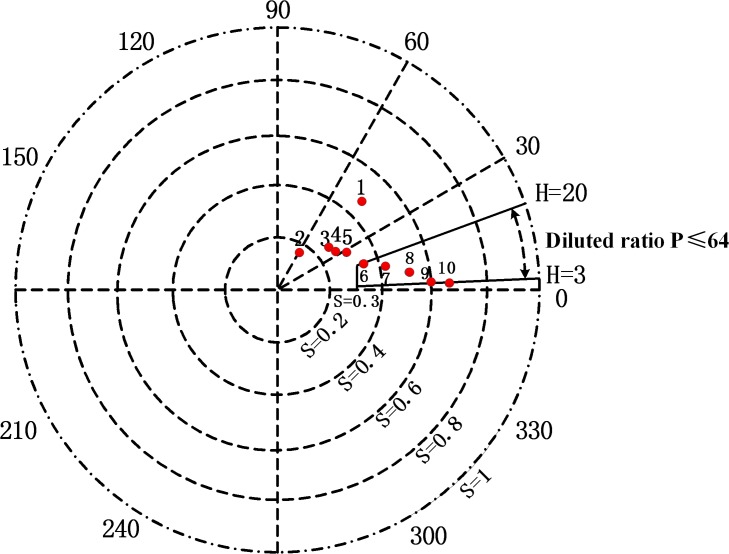
HS distribution corresponding to different samples.

According to Eqs (4) and (5), where *m* is the maximum value of RGB and *n* is the minimum value of RGB, the color characteristic values of H and S can be obtained. H and S values of each sample solution are shown in Table 1, and the distribution of H and S is shown in Fig 7.

$$S=\{\begin{array}{c} \frac{m-n}{m+n}\\ \frac{m-n}{510-(m+n)} \end{array}\begin{array}{cc} & \begin{array}{c} \left(L<128\right)\\ \left(L\geq 128\right) \end{array} \end{array}$$ (4)

$$H=\{\begin{array}{c} 60[0+(G-B)/(m-n)]\\ 60[2+(B-R)/(m-n)]\\ 60[4+(R-G)/(m-n)] \end{array}\begin{array}{c} \left(R=m\right)\\ \left(G=m\right)\\ \left(B=m\right) \end{array}$$ (5)

As shown in Fig 7, the less the sample solution was diluted, the higher its hemoglobin concentration and the deeper its red color (and the smaller the corresponding H value, the greater the S value.) At sample dilution ratio P≤64 and hemoglobin concentration C≥152mg/ml/64 = 2.375mg/ml, color characteristic values H and S were in ranges H∈[3,20], S∈[0.33,0.8]. In actuality, for patients with intestinal bleeding, hemoglobin concentration in bleeding areas is higher than 2.375mg/ml [37]; the corresponding characteristic H and S values of the mixture of intestinal juice and blood in bleeding regions indeed fall within this range. To ensure that even relatively small amounts of intestinal bleeding were registered appropriately by the system, we set a higher threshold hue H1 = 25 and a lower saturation S1 = 0.3. H∈[3, H1] and S∈[S1,0.8] were the final bleeding recognition conditions.

Test results of the experiment to verify the wireless data transmitting and receiving capabilities of the IBDC system, as well as the effectiveness of the bleeding alarm are shown in Table 2.

**Table 2:** Status of buzzer and LED under different experimental conditions.

Solution No.	1	2	3
**Buzzer (sound or not)**	not	not	sound
**LED (light or not)**	not	not	light

Only Solution 3 triggered the alarm during the experiment (Table 2), indicating that bleeding detection module can work and trigger the wireless transmitter module effectively. The wireless transmitting and receiving modules worked normally, and the alarm module can alarm correctly when bleeding in the simulation experiment. Since the operating voltage had been above the threshold, the buzzer didn’t sound in Solution 1 and Solution 2 tests.

### 3.2 System Power Consumption Evaluation

The wireless transmitter module can only be triggered when bleeding symptoms are recognized, at which point it instantaneously emits necessary data to the receiving module. The energy consumed by this process is very low. The wireless transmitter module is in the off state, with almost zero energy consumption, when not being triggered. At 3V operating voltage, the color sensor’s operating current is about 2mA and the lighting circuit’s current is about 5mA. The working current of the microcontroller is about 0.6mA. As a result, the current of the intestinal bleeding intelligent detection capsule is near 7.6mA while the capsule is working. In the dormant period, all modules within the capsule are closed except the watchdog timer of the microcontroller (the operating current of which approximates to zero). In the process of in vivo detection, the capsule adopts an intermittent functionality, working for 0.5s and sleeping for 5s. The average working current is about 0.7mA and average power consumption is estimated to be about 2.1mW.

## 4. Conclusions

The intelligent IBDC system proposed in this paper was designed to recognize intestinal bleeding automatically according to a set range of H and S values in HSL color space, which was consistent with human visual characteristics. A bleeding detection module, a wireless transmission module, a wireless receiving module and an alarm module were designed and verified to work effectively. The IBDC system can work effectively and recognize bleeding in the simulated experiment. The design of the intermittent working mode realized low power consumption after the integration of functional modules. Bleeding recognition conditions were set as 3≤H≤25 and 0.3≤S≤0.8, and if H and S met the set conditions, it indicated the existence of intestinal bleeding in the in vitro scenario.

The smart IBDC system is noninvasive, simple, and greatly reduces the workload on physicians on account of automatic alarm. When the system recognizes intestinal bleeding, the in vitro bleeding position (and thus, confirmed the diagnosis) of the patient is provided near instantaneously, allowing the physician to feasibly and effectively set the course of treatment. The next step of the study will be to add drug release functionality, test specific diagnoses for trace, small, or large amounts of blood. We expect that the smart capsule system represents the next generation of intestinal bleeding diagnosis devices.

## Abbreviations

IBDC: intelligent intestinal bleeding detection capsule
HSL: hue-saturation-light
GI: gastrointestinal
LED: light emitting diode
RGB: red-green-blue
RF: radio frequency
QFN: Quad Flat No-lead Package
ISM: Industrial Scientific Medical
SMD: Surface Mounted Devices
